# Intensity-Dependent Effect of Treadmill Running on Knee Articular Cartilage in a Rat Model

**DOI:** 10.1155/2013/172392

**Published:** 2013-12-31

**Authors:** Guo-Xin Ni, Sheng-Yao Liu, Lei Lei, Zhe Li, Yue-Zhu Zhou, Li-Qiong Zhan

**Affiliations:** ^1^Department of Orthopaedics and Traumatology, Nanfang Hospital, Southern Medical University, 1838 Guangzhou Avenue (N), Guangzhou 510515, China; ^2^Department of Rehabilitation Medicine, Longyan People's Hospital, 31 Denggao Road (W), Longyan 364000, China; ^3^Department of Rehabilitation Medicine, 1st Affiliated Hospital, Fujian Medical University, 20 Chazhong Road, Fuzhou 350005, China

## Abstract

*Objective*. To understand the changes of femoral cartilage in response to treadmill running with different intensities in the hope of differentiating “moderate” and “strenuous” running in a rat model. *Method*. A total of 24 male Wistar rats were randomly assigned into groups of sedentary (SED), low-intensity running (LIR), medium-intensity running (MIR), and high-intensity running (HIR). Rats in LIR, MIR, and HIR groups underwent 8 weeks' treadmill running programs. After sacrificed, femoral condyles were collected to take histomorphometric analysis and immunohistochemistry for collagen II. *Results*. Gross and histological observation showed osteoarthritic changes in group HIR. In comparison to SED group, there was significant increase in cartilage thickness, number of chondrocytes, and GAG content in groups LIR and MIR. Conversely, decrease in cartilage thickness, chondrocyte number, and GAG content was found in rats of HIR group, without significant difference though. In addition, in comparison to SED group, HIR group exhibited disorganization of collagen fibril and significantly lower content of collagen type II. *Conclusion*. An intensity-dependent effect was suggested on the articular cartilage. Our results also demonstrated that running with low-to-medium intensity applied in the present study should be regarded as “moderate” running, whereas high-intensity running as “strenuous” running.

## 1. Introduction

Osteoarthritis (OA) is a progressive degenerate joint disease that affects the structural and functional integrity of joint tissues, which is mainly characterized by cartilage degradation. The biosynthetic activity of chondrocytes is responsive to mechanical stimuli and can alter the morphology and composition of cartilage [1]. It is now generally agreed that reduced loading has deleterious effects on articular cartilage [2]. Undoubtedly, a certain amount of joint loading is needed to maintain normal cartilage properties. However, excessive loading may have deleterious effects.

Regular exercise is considered an integral component of a healthy lifestyle; however, its effect on articular cartilage remains a subject of debate and speculation, as does the relationship of exercise to the development of OA [3]. Taking running, one of the most common weight-bearing activities, for example, its effect on the cartilage of knee joint appears conflicting. Findings from some studies using animal models suggested that running exercise may be beneficial to the cartilage health [4, 5], while others demonstrated deleterious effect [6–8]. A general explanation to these inconsistent results is that “moderate” running program is related to the beneficial effect, whereas “vigorous” or “strenuous” running to the deleterious effect. “Strenuous” running is regarded as a reliable OA animal model [8].

Nevertheless, the problem lies in how to differentiate the so-called “moderate” and “strenuous” running program, thereby creating a reliable running-induced OA animal model and guiding the running exercise. Needless to say, due to different exercise capacity among animal species, it is difficult to extrapolate findings from one animal model to others or human beings. Even for the same animal model, varied running programs lead to the obvious difficulty of correlating the data [6–9]. For example, rats running 500 m/day in a running wheel for 12 weeks showed local softening of the articular cartilage and decreased glycosaminoglycans (GAG) [7]. However, in another rat model, “strenuous” running program was defined as a total of 30 km running distance in 6 weeks with gradual increase in running speed and time [9]. Clearly, no consensus has been made with respect to the definition of “strenuous” running program.

Moreover, without differentiating “moderate” and “strenuous” running, it is impossible to efficiently guide the running training, which may provide many benefits physically and emotionally. Dosage-dependent response of running was previously suggested to cartilage of rats subjected to anterior cruciate ligament transaction [10, 11]. However, such response to intact cartilage remains unclear. A common way to normalise exercise intensity is to express it as the percentage of VO_2max_, which is generally considered the best indicator of cardiorespiratory endurance and aerobic fitness [12]. Evidence showed that the higher the exercise intensity, the greater the increase in aerobic fitness [13, 14]. It appears that more vigorous exercise has greater cardioprotective benefits. Nevertheless, such intensity-dependent effect should also be investigated on other systems or organs for a comprehensive evaluation so as to determine the “appropriate” running intensity.

Previously, for research purposes, Bedford et al. [15] demonstrated the respective percentage of VO_2max_ related to various treadmill running programs for Wistar rat. Therefore, in the present study, three treadmill running programs were selected to elicit the low, medium, and high intensity with about 60%, 75%, and 90% VO_2max_ values, respectively, and their respective effects were investigated on articular cartilage of knee in order to differentiate “moderate” and “strenuous” running in a rat model.

## 2. Materials and Methods

### 2.1. Experimental Animals and Exercise Protocols

A total of 24 male Wistar rats (12-13 weeks old, weight 200–250 g) were randomly and evenly assigned to one of four groups as follows: (1) sedentary control (SED, *n* = 6), (2) low-intensity running (LIR, *n* = 6), (3) medium-intensity running (MIR, *n* = 6), and (4) high-intensity running (HIR, *n* = 6). All animals were housed in cages under controlled light/dark (12/12 h) and temperature (22 ± 1°C) conditions and provided with food and water ad libitum. This study was approved by the animal ethics committee of the institute.

All animal were firstly accustomed to exercise for 1 week, by running on a treadmill at speed of 10 m/min for 30 min/day. Subsequently, animals in LIR, MIR, and HIR groups were regularly trained according to the following running protocols for 8 weeks, which were used to elicit the low (~60% VO_2max_), medium (~75% VO_2max_), and high intensity (~90% VO_2max_) for Wistar rats, respectively [15]. Meanwhile, rats in group SED, which were maintained in cages without any additional exercise, serve as control. All experiments were conducted in accordance with our institutional guidelines for the care and use of experimental animals as follows:

- LIR: speed of 15.2 m/min with inclination (0°) for 60 min, 5 days/week,
- MIR: speed of 19.3 m/min with inclination (5°) for 60 min, 5 days/week,
- HIR: speed of 26.8 m/min with inclination (10°) for 60 min, 5 days/week.

### 2.2. Tissue Preparation

At the end of 8 weeks' running program, all animals were sacrificed. The right knee joints were disarticulated and the surrounding soft tissue was removed. Femoral condyles of the medial compartment in each group were dissected and fixed in 4% buffered formaldehyde pH 7.4 for 24 hours. Decalcification was completed in 10% EDTA solution, and then the samples were embedded in paraffin wax. Thereafter, they were cut into 5 *μ*m sagittal sections in the medial region for further investigations.

### 2.3. Histomorphological Evaluation

The samples were stained with hematoxylin eosin (HE), Safranin-O, and Picrosirius red for histological observation, respectively. Histomorphometric analyses were taken on HE-stained sections using method described elsewhere [16]. In short, for each section, thickness from subchondral bone to articular surface was measured at three sites, that is, the center of articular surface, 300 *μ*m left and right, respectively, and then averaged. In each section, chondrocyte cells were counted within a 120,000 *μ*m^2^ area including calcified layer and articular surface. Each section was evaluated by two blinded observers and then correlated.

The sections stained with Safranin-O were examined by Nikon H600L Microscope with an image analysis system (Nikon, Japan). GAG content was evaluated in Safranin-O-stained sections using digital densitometry. For each section, six different areas were digitally captured with a colour video camera attached to a light microscope (Nikon H600L Microscope and image analysis system, Japan). Illumination intensity and image magnification were kept constant for all images captured. The information was assessed with computer image analysis software. The values of optical density in six areas were averaged to be GAG content in each section.

Polarizing light microscopy was used to evaluate the alignment of collagen fibers in Picrosirius red-stained sections. The sections were examined under optical microscope equipped with a polarizing light coupled to an image analysis system (Olympus CCD DP71/Olympus Microscope BX-51).

### 2.4. Immunohistochemistry for Collagen Type II

In addition to histomorphological evaluation, immunohistological analysis for collagen type II was performed in all sections. After deparaffinization and rehydration of the tissue sections, collagen type II was immunostained with the two-step immunohistochemistry method instructed by the manufacturer (Zhongshan Goldenbridge Biotechnology Co., Ltd., China).

The sections were incubated with monoclonal mouse anti-rat collagen type II antibody (1 : 200 dilution, Fisher Scientific, IL, USA) for 3.5 h at 29°C. The slides were washed in PBS for 3 times, followed by a 20 min incubation at 37°C with anti-mouse IgG/HRP (Fisher Scientific, IL, USA), and visualized with DAB chromagen. Thereafter, the nucleus was counterstained with hematoxylin for 6 s. Negative control sections were prepared with the same protocol above, but primary antibody was replaced by PBS. The collagen type II content was evaluated based on optical density measured using image analysis software (Nikon H600L Microscope and image analysis system, Japan).

### 2.5. Statistical Analysis

Results are expressed as the mean ± standard deviation. Statistical analysis was carried out using a one-way ANOVA and Tukey's test for post hoc analysis with significance set at *P* < 0.05.

## 3. Results

**Figure 1:**
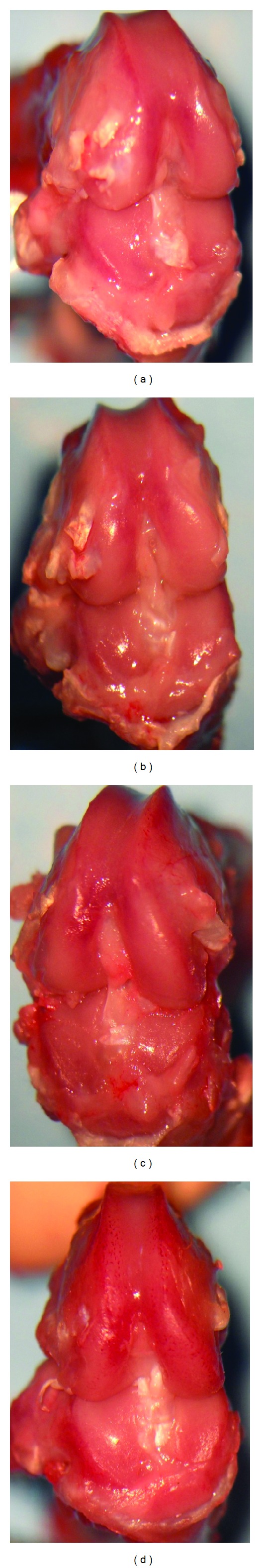
Macroscopic view of the surfaces of femoral articular cartilage in SED, LIR, MIR, and HIR groups. Smooth and glistening surface was observed in group SED (a). Similar appearance of the cartilage was found in groups LIR (b) and MIR (c). However, the cartilage surface in group HIR became lusterless and rough (d).

From gross observation, the surface of the femoral articular cartilage in group SED was smooth and glistening (Figure 1(a)). Similar appearance of the cartilage was observed in groups LIR (Figure 1(b)) and MIR (Figure 1(c)). However, the cartilage surface in group HIR became lusterless and rough (Figure 1(d)).

**Figure 2:**
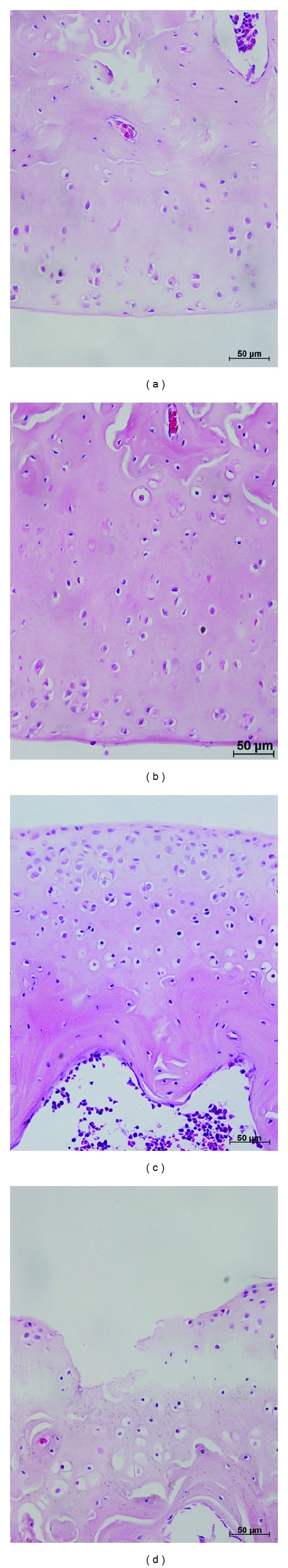
Histological morphology of femoral articular cartilage with hematoxylin eosin staining in SED (a), LIR (b), MIR (c), and HIR (d) groups. Grossly normal histological characteristics of cartilage sections were observed in groups SED, LIR, and MIR. In contrast, osteoarthritic histological changes of surface irregularities and cell cloning were found in group HIR.

**Figure 3:**
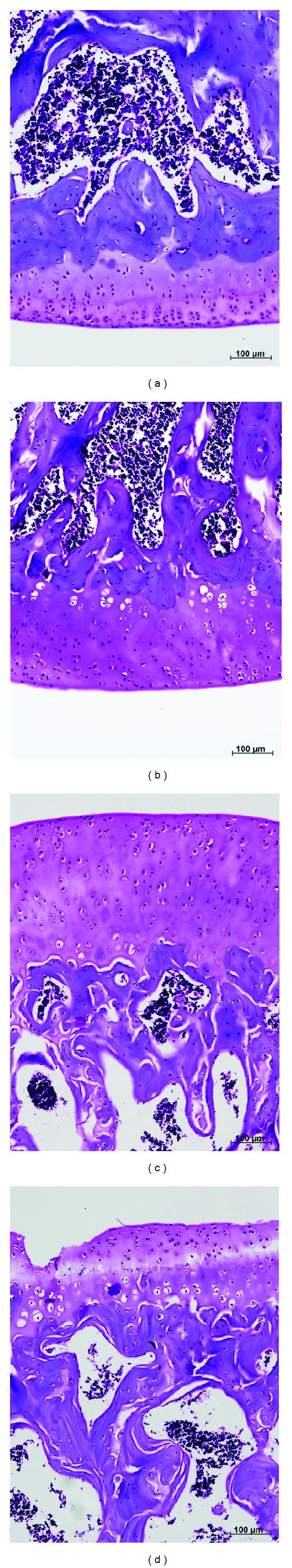
Histological morphology of femoral articular cartilage with Safranin-O staining in SED (a), LIR (b), MIR (c), and HIR (d) groups. Although grossly normal histological characteristics of cartilage sections were observed in SED, LIR, and MIR groups, the increased staining for Safranin-O was clearly found in LIR and MIR groups in comparison with SED group. In contrast, osteoarthritic histological changes of surface irregularities, cell cloning, and moderate reduction in the safranin-O staining were found in HIR group.

Grossly normal histological characteristics of cartilage sections were observed in groups SED, LIR, and MIR. In contrast, histological changes of surface irregularities, cell cloning, and moderate reduction in the Safranin-O staining were found in group HIR (Figures 2 and 3).

Histomorphometric analyses were taken on HE-stained sections in four groups to obtain the cartilage thickness, chondrocyte number, and GAG content (Table 1). Significant increase in cartilage thickness was found in groups LIR and MIR, as compared to group SED. No significant difference was between groups SED and HIR. However, the cartilage thickness in group HIR was significantly lower than that in LIR or MIR group. Similar changing pattern was observed for number of chondrocytes and GAG content. There were significantly more chondrocytes in group LIR or MIR than group SED. Chondrocytes in group HIR were significantly less than those in LIR or MIR group. However, no significant difference existed between HIR group and SED group. Compared to group SED, significantly higher GAG content was found in groups LIR and MIR, respectively. The GAG content in HIR group was lower than that in SED group, without statistical difference between them.

**Figure 4:**
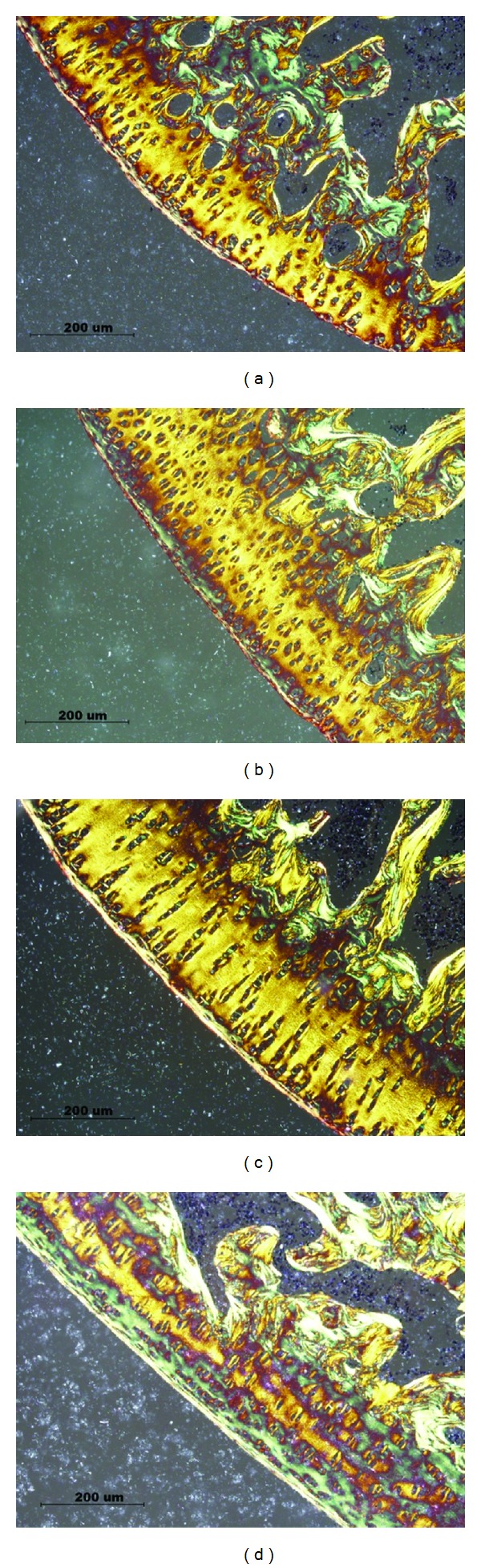
Photomicrograph of the articular cartilage with Picrosirius red staining in SED (a), LIR (b), MIR (c), and HIR (d) groups. Collagen fibers in groups LIR and MIR exhibit the same organization as shown in group SED. However, disorganization of collagen fibers was observed in group HIR.

**Figure 5:**
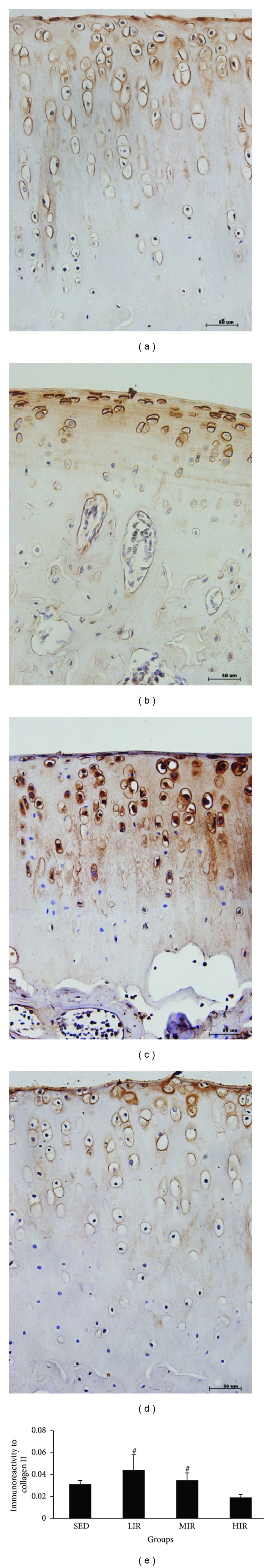
Immunostaining of collagen II of femoral articular cartilage in SED (a), LIR (b), MIR (c), and HIR (d) groups. In comparison to group SED, increased staining of collagen II was found in LIR and MIR groups, whereas less staining was detected in HIR group. Collagen II content in each group was shown in (e). In comparison with group SED, higher collagen content was found in groups LIR and MIR, without significant difference. However, the collagen content in group HIR was significantly lower than that in group SED. ^#^ *P* < 0.05 compared to HIR group.

Figure 4 showed the images of the histological sections of four groups stained with Picrosirius red. It appears that the collagen fibers in groups LIR and MIR exhibit the same organization as shown in group SED. However, disorganization of collagen fibers was observed in group HIR. Immunohistological analysis for collagen type II was performed in all sections, and the content of collagen type II in each group was presented in Figure 5. In comparison with group SED (0.0309 ± 0.0036), no significant difference was found in collagen content in group LIR (0.0437 ± 0.0144) or group MIR (0.0344 ± 0.0071). However, the collagen content in group HIR (0.0188 ± 0.003) was significantly lower than that in group SED.

## 4. Discussion

Articular cartilage is mechanoadaptive; that is, the biosynthetic activity of chondrocytes is responsive to mechanical stimuli and can alter the morphology and composition of cartilage [1]. Physical loading is regarded as a two-sided sword; however, it remains unclear of the “appropriate” running-induced joint loading [17]. In the present study, an intensity-dependent effect of running was demonstrated on the articular cartilage. It was also suggested that running with low-to-medium intensity would maintain the integrity of cartilage and should be regarded as “moderate” running, whereas high-intensity running would damage articular cartilage and should be regarded as “strenuous” running.

In the literature, numerous animal OA models have been reported [18]. Since OA is thought to be a multifactorial disease, it is necessary to use multiple murine models to best understand OA disease progression. Among them, exercise-induced animal model is considered as a more reliable OA model than genetically, surgically, or chemically induced model [8, 19], since OA occurs without a definite mutation or history of injury. It was proposed that there is a certain range of loading of cartilage that is conducive to its health; above and below this the tissues suffer [20]. However, how to define the safe upper limit for such stress for cartilage is still a matter of question. So far, direct measurements of *in vivo* cartilage-on-cartilage contact stresses due to overuse in human joints have not been made. The *in vitro* results of the range of nonphysiological loading intensities cannot exactly extrapolate to the *in vivo* situation. Therefore, in practice, how to define the range of physiological loading remains an immediate concern. Using the same animal model, we previously demonstrated an intensity-dependent effect of treadmill running on lubricin metabolism of rat articular cartilage [21]. Our findings in the present study further demonstrated different responses of cartilage to different running intensities, indicating that the running loads may be within and above the safe upper limit during low-to-medium intensity and high-intensity running, respectively.

The articular cartilage consists of chondrocytes embedded in an abundant extracellular matrix composed primarily of type II collagen and the proteoglycan aggrecan. In the present study, running with low-to-medium intensity leads to a significant increase in cartilage thickness, number of chondrocytes, and GAG content, as well as an increase in collagen content, suggestive of beneficial effect on cartilage integrity. “Moderate” running was previously reported to improve cartilage GAG content in dog [4] and human [22]. As the only cell type of the articular cartilage, chondrocytes are responsible for the synthesis and maintenance of the extracellular matrix and can react to mechanical forces and structural changes in the extracellular matrix [23]. Antiapoptotic effects were ever suggested by “moderate” running on cartilage of experimental OA rats [10, 24]. It is supposed that “moderate” running may lead to antiapoptotic effect on intact cartilage and increased number of chondrocytes as a result. The resultant increase in GAG content would improve the viscoelasticity of cartilage and protect the collagen network from compressive forces.

Conversely, group HIR exhibited decreased cartilage thickness, chondrocyte number, and GAG content than group SED, without significant difference though. Notably, in comparison to group SED, group HIR showed disorganization of collagen fibril and significantly lower content of collagen type II. Similar results were previously reported on beagle femoral cartilage after running exercise of 20 km/d or 40 km/d for up to 15 weeks [25, 26]. Loss of proteoglycans and the breakdown of the cartilage collagen network are typical of osteoarthritic cartilage [27]. Obviously, in the present study, the high-intensity running-induced loading on cartilage, which results in OA-like changes, is above the safe upper limit. Excessive mechanical stress can directly damage the cartilage extracellular matrix and shift the balance in chondrocytes to favor catabolic activity over anabolism [28], leading to the degradation of both collagen fibrils and proteoglycans [29]. This involves a variety of degradative enzymes, including matrix metalloproteinases (MMPs) [6].

Running is an excellent activity to promote general health and well-being. However, it remains unknown as to whether “strenuous” running is a predisposing cause of osteoarthritis or not [3]. Individual variation in response to exercise may be a key factor to account for the conflicting findings in the literature [30]. Therefore, the so-called “strenuous” running should be defined individually. In this sense, to differentiate “moderate” from “strenuous” running, intensity may be better expressed as percentage of VO_2max_, as did in the present study, than as running distance and speed alone. However, the relationship between running intensity and the induced loading on cartilage should be clarified in the future study. The so-called “moderate” and “strenuous” running should induce running loads within and above the safe upper limit to cartilaginous tissue, respectively. For one thing, our study only addressed the responses of three running programs for Wistar rat. Although our results showed that running with low-to-medium was regarded as “moderate” running and high-intensity running as “strenuous” running, it should be noted that the same percentage of VO_2max_ could be obtained by adjusting running speed and inclination, leading to different mechanical loadings on cartilage. What is more, caution should be taken to extrapolate our results to human beings. Therefore, future investigations should be taken to address these concerns.

## 5. Conclusion

Regular aerobic exercise training brings about a number of bodily changes that benefit the entire organism in general. However, the optimum “dose” of exercise is not certain. Clearly, a comprehensive evaluation should be taken for “appropriate” exercise intensity on various systems. Our study, for the first time, investigated the effect of running intensity, expressed as percentage of VO_2max_, and an intensity-dependent effect was suggested on the articular cartilage. It was also shown that running with low-to-medium intensity should be regarded as “moderate” running, whereas high-intensity running applied in the present study should be regarded as “strenuous” running. More studies are needed to convince that such “strenuous” exercise could be used to create a reliable running-induced OA animal model.

## Figures and Tables

**Table 1:** Cartilage thickness, chondrocyte number, and GAG content in four groups.

	SED	LIR	MIR	HIR
Cartilage thickness (*μ*m)	171.61 ± 52.19	210.88 ± 55.71^∗#^	232.47 ± 65.39^∗#^	161.65 ± 43.39
Chondrocyte number	88.67 ± 23.62	149.67 ± 28.99^∗#^	130.67 ± 10.23^∗#^	73.17 ± 9.66
GAG content	0.086 ± 0.016	0.134 ± 0.032*	0.138 ± 0.023*	0.077 ± 0.019

*P < 0.05 compared to SED group; ^#^ *P* < 0.05 compared to HIR group.

## Abbreviations

OA: Osteoarthritis
SED: Sedentary
GAG: Glycosaminoglycans
HE: Hematoxylin eosin
LIR: Low-intensity running
MIR: Medium-intensity running
HIR: High-intensity running
RT-PCR: Real-time polymerase chain reaction.
